# Brain activation during visual working memory correlates with behavioral mobility performance in older adults

**DOI:** 10.3389/fnagi.2015.00186

**Published:** 2015-09-29

**Authors:** Toshikazu Kawagoe, Maki Suzuki, Shu Nishiguchi, Nobuhito Abe, Yuki Otsuka, Ryusuke Nakai, Minoru Yamada, Sakiko Yoshikawa, Kaoru Sekiyama

**Affiliations:** ^1^Graduate School of Social and Cultural Sciences, Kumamoto UniversityKumamoto, Japan; ^2^Japan Society for the Promotion of ScienceTokyo, Japan; ^3^Faculty of Letters, Kumamoto UniversityKumamoto, Japan; ^4^Graduate School of Medicine, Kyoto UniversityKyoto, Japan; ^5^Kokoro Research Center, Kyoto UniversityKyoto, Japan

**Keywords:** working memory, functional mobility, aging, compensation, age-related change, fMRI

## Abstract

Functional mobility and cognitive function often decline with age. We previously found that functional mobility as measured by the Timed Up and Go Test (TUG) was associated with cognitive performance for visually-encoded (i.e., for location and face) working memory (WM) in older adults. This suggests a common neural basis between TUG and visual WM. To elucidate this relationship further, the present study aimed to examine the neural basis for the WM-mobility association. In accordance with the well-known neural compensation model in aging, we hypothesized that “attentional” brain activation for easy WM would increase in participants with lower mobility. The data from 32 healthy older adults were analyzed, including brain activation during easy WM tasks via functional Magnetic Resonance Imaging (fMRI) and mobility performance via both TUG and a simple walking test. WM performance was significantly correlated with TUG but not with simple walking. Some prefrontal brain activations during WM were negatively correlated with TUG performance, while positive correlations were found in subcortical structures including the thalamus, putamen and cerebellum. Moreover, activation of the subcortical regions was significantly correlated with WM performance, with less activation for lower WM performers. These results indicate that older adults with lower mobility used more cortical (frontal) and fewer subcortical resources for easy WM tasks. To date, the frontal compensation has been proposed separately in the motor and cognitive domains, which have been assumed to compensate for dysfunction of the other brain areas; however, such dysfunction was less clear in previous studies. The present study observed such dysfunction as degraded activation associated with lower performance, which was found in the subcortical regions. We conclude that a common dysfunction—compensation activation pattern is likely the neural basis for the association between visual WM and functional mobility.

## Introduction

Previous studies have indicated that physical exercise is beneficial to cognitive functions in older adults (Colcombe et al., 2004; Kramer et al., 2006; Larson et al., 2006; Hillman et al., 2008; Deary et al., 2009; Smith et al., 2010). Among the many kinds of cognitive function, executive function has most firmly been shown to improve through exercise (Colcombe and Kramer, 2003). Executive function is a high level mental process which is thought to be heavily involved in handling novel situations outside the domain of our automatic psychological processes with goal-directed behavior (Banich, 2009). Based on the previous studies suggesting that executive function is deeply overlapped with working memory (WM; Baddeley, 2000, 2012; Miyake et al., 2000; D’Esposito, 2007; Friedman et al., 2008; McCabe et al., 2010), Kawagoe and Sekiyama (2014) investigated the relationship between WM and functional mobility. WM is thought to have at least two subcomponents, verbal-based and visual-based, (Baddeley, 1986, 2000, 2012). The substantial differences of those components have been shown (Smith and Jonides, 1997), as has these types differing vulnerability to aging (Jenkins et al., 2000; Leonards et al., 2002). We demonstrated that visually encoded WM (i.e., WMs for location and face stimuli) was related to functional mobility, which needed dynamic motor controls, while verbally encoded WM was not (Kawagoe and Sekiyama, 2014). In that study, WM was assessed by an N-back task and mobility was assessed by a Timed Up and Go Test (TUG; Podsiadlo and Richardson, 1991), which is one of the major clinical assessments of functional mobility in older adults.

The present study aimed to elucidate the neural correlates of this WM-mobility association in older adults using functional Magnetic Resonance Imaging (fMRI). Brain activation during physical movement has been recently explored in the comparison between younger and older adults. For example, they used a cyclical hand and foot flexion and extension task across different degrees of complexity (Heuninckx et al., 2005) and a controlling grip force magnitude task (Noble et al., 2011). By and large, however, the brain activations during locomotion are difficult to explore with the exception of the [^18^F]-FDG PET technique (la Fougère et al., 2010). We used another approach to assess the neural basis of the association between WM and mobility. Based on the brain—behavior relationship repeatedly confirmed as a correlation between brain activation and behavioral performance (Todd and Marois, 2004; Davis et al., 2008; Berchicci et al., 2014), and on the significant correlation between WM and mobility at a behavioral level (Kawagoe and Sekiyama, 2014), we focused on the association between brain activations during WM and behavioral mobility performance.

A common neural basis for WM and mobility were anticipated in the prefrontal and parietal (i.e., attentional) regions because both of those are certainly needed for WM and mobility especially in older adults. The frontal regions are involved in WM at the monitoring status (Petrides, 1991) and the parietal regions contribute to manipulation of information in WM (Champod and Petrides, 2007) although the specific roles and their relationship were still controversial. At any rate, those regions are the main areas involved in WM (Collette et al., 2005; Owen et al., 2005; Rottschy et al., 2012), and named as the fronto-parietal network. Correspondingly, mobility control may involve those regions. Although mobility control basically does not need the prefrontal regions in younger adults (la Fougère et al., 2010: primary areas such as M1 and S1 are more important for younger adults), older adults need more prefrontal recruitment for physical movements (Heuninckx et al., 2005, 2008; Blumen et al., 2014), even for simple motor response (Berchicci et al., 2014) and physical force control (Noble et al., 2011) than younger adults. Such prefrontal involvement (overrecruitment) is conceptualized as “compensation”, which is based on the assumption that the overrecruitment would compensate for the deterioration of other brain regions. Moreover, both the parietal and prefrontal areas activation during a cognitive task might be related to levels of aerobic fitness in older adults (Colcombe et al., 2004). Thus, it is possible that a WM-mobility association could be observed in the prefrontal and parietal regions in older adults.

Although different views have been postulated for overrecruitment in older adults (Lindenberger and Baltes, 1994), compensation is likely to be the case when there are no differences in behavioral performance between younger and older adults (Heuninckx et al., 2008; Eyler et al., 2011). The compensation refers to processing inefficiencies that cause the older brain to recruit more neural resources to achieve a computational output equivalent to that of a younger brain. One influential model of compensation, the Compensation-Related Utilization of Neural Circuits Hypothesis (CRUNCH) postulates that the compensatory activation is effective at lower levels of task demand, but as demand increases, a resource ceiling is reached, leading to insufficient processing and age-related degraded performance for harder tasks (Reuter-Lorenz and Cappell, 2008). In consistent with the CRUNCH, prior studies have indicated that good cognitive performers are able to carry out an experimental easy task more efficiently (with lower levels of brain activation) than low performers (Smith et al., 2001; Daffner et al., 2011). This kind of compensation would be true for motor control because the athlete (Naito and Hirose, 2014) and older adults who exercise (Berchicci et al., 2014) could achieve simple motor tasks more efficiently than controlled counterparts, and as the task difficulty increases, more increased brain activation was observed (Winstein et al., 1997). By applying the concept of that model to the WM-mobility association within healthy older adults, when the WM task requires a low cognitive load, the activation level of the regions activated by the cognitive task would be negatively correlated with the mobility performance (thus positively correlated with the time needed to complete TUG in this study). To this end, the WM task in the fMRI was set to be as easy as possible so that we were able to make a clear interpretation of older adults’ brain activation, such as the poor mobility performer would activate some brain regions more than the good mobility performer for an equivalent level of visual WM performance. We anticipated that such brain-behavior correlation also would be found in the context of WM-mobility association.

Based on CRUNCH, we expected that poor mobility performers activate more prefrontal and parietal regions than good performers when the WM task is cognitively low-demand because we already demonstrated the behavioral correlation between visual WM and mobility (Kawagoe and Sekiyama, 2014). If there were a close relationship between WM and TUG, we would find the correlation in the executive-related attentional regions including the prefrontal and parietal areas. That kind of correlation would indicate that the compensatory activation in the prefrontal and parietal areas is a common mechanism against age-related decline, and that activation might be the reason for the significant relationship between cognitive and motor performance in older adults. We mention that TUG was used for the present study because we had already found a relation to WM, but a simple walking test was also measured in this study because that has been suggested as an important geriatric measure in older adults (Yogev-Seligmann et al., 2008; Bohannon and Williams Andrews, 2011; Peel et al., 2013). However, as shown below, there was no substantial correlation between simple walking and the WM task.

To summarize, the present study aimed to uncover the neural basis for the association between visually encoded WM and mobility. Our hypothesis was that levels of brain activations of WM, perhaps in prefrontal and parietal parts, would be significantly correlated with mobility performance. Such neural correlation would reveal the reason why there is a significant behavioral relationship between WM and mobility in older adults.

## Materials and Methods

### Participants

Participants were recruited through Kyoto City Silver Human Resources Centre of 32 Japanese older adults aged 60 years or older living in Kyoto city (age: 73.06, SD:4.83yrs). Mini-Mental State Examination (MMSE; Folstein et al., 1975) and a set of interviews was performed to exclude participants based on the following exclusion criteria: a score of less than 27 on the MMSE, a history of major psychiatric illness, serious neurological diagnoses, severe cardiac, pulmonary, or musculoskeletal disorders and use of psychotropic drugs. We established a slightly strict standard for the MMSE cut-off point to better enable the analysis to eliminate the effect of general cognitive decline (Rajah et al., 2010). The study protocol was approved by the ethical committee of Kyoto University Graduate School of Medicine. Written informed consent was obtained from each participant for the trial in accordance with the Helsinki Declaration of Human Rights.

### fMRI Task

The participant was asked to perform N-back tasks for location and face stimuli during fMRI scanning. In the present study, we used only 1-back task as a WM task for the interpretation of brain activations on the basis of CRUNCH. The 0-back task and rest conditions were conducted as baseline or control tasks. In the 1-back task, participants were asked to monitor a series of stimuli (single dot location or face) and to indicate whether the stimulus was the same as the one presented in the previous trial and press one of two buttons with the right hand, pressing the left button with an index finger for “same” response and right button with a middle finger for “not same” response. In the face 0-back task, participants were asked to indicate the gender of a face stimulus. In the location 0-back task, participants were required to indicate whether or not it was located in the center of the screen. There was also a rest condition in which participants were asked to gaze at a fixation cross located in the center of the screen. The location WM task and face WM task were conducted in separate fMRI runs. The location stimuli were made up of a single black dot, which was presented in a randomly designated location determined by vertical and horizontal coordinates. The face stimuli were made up of the neutral faces of Japanese young people (university students), with an equal number (26) of male and female faces. Face and location stimuli were not shown in a repetitive fashion except the stimuli for “hit” in the 1-back task, and the centered-dot, which needed a “hit” in the 0-back task for location. The rates of “hit” for 0- and 1-back tasks was 37%. Participants were not requested to respond as fast as possible but the stimulus duration was set as two seconds and the SOA (stimulus onset asynchrony) as four seconds. The blank image was presented between the stimuli. If the participant could not make any response until the next stimulus presentation, the response was recorded as a miss. Each of the three conditions (1-, 0-back tasks, and rest) was conducted in separate blocks. Each block, which had a duration of 32 s (i.e., 8 trials for 1- and 0-back tasks), was presented four times (a total of 12 blocks) and there were an equal number of each condition within the block. For both the face and location WM tasks, the order of the 12 blocks was counterbalanced across participants. Before the fMRI scanning, the participant was given practice trials outside the magnet with stimuli different from the test stimuli.

### fMRI Data Acquisition

Whole-brain imaging was performed using a 3.0-Tesla Siemens Magnetom Verio MRI scanner. A T2*-weighted echo planar imaging (EPI) sequence sensitive to blood oxygenation level-dependent (BOLD) contrast. The following parameters were used for functional imaging: repetition time (TR) = 2000 ms, echo time (TE) = 25 ms, flip angle = 75°, acquisition matrix = 64 × 64, field of view (FOV) = 224 mm, in-plane resolution = 3.5 mm × 3.5 mm, and 39 axial slices with a slice thickness of 3.5 mm. A high-resolution structural image was also acquired using a T1-weighted magnetization prepared rapid-acquisition gradient echo (MP-RAGE) pulse sequence (voxel size = 1 × 1 × 1 mm^3^). Firm padding was placed around the head of each participant to restrict head motion. Stimuli were projected onto a screen viewed by the participant via a mirror mounted on the scanner head coil and the participants’ responses were collected using a MRI-compatible response box. The EPI images were acquired in two consecutive runs (197 volumes per run; i.e., one for face WM task and the other for location WM task, see below). The first five scans in each run were discarded to allow for T1 equilibration effects.

### Mobility Task

Two mobility tests were set up. TUG was used as the index of their functional mobility (Podsiadlo and Richardson, 1991) and a 10 m walking test (10MWT) as a simple walking measurement. In the TUG, participants were instructed to stand up from a standard chair, walk a distance of 3 m, turn, walk back to the chair, and sit down. Participants were asked to complete it as quickly as possible. In the 10MWT, participants walked over a distance of 10 m, at their maximum speed. The performed times were measured by the experimenter with a stop watch.

### Pre-Processing and Statistical Analysis

#### Behavioral Data

First, the WM performances were compared by a 2 (cognitive load: 0- and 1-back) × 2 (stimuli: face and location) ANOVA. Then, we conducted correlation analyses for the WM and mobility performances. Because the distribution for normality was not confirmed (the skewedness was tested by a Kolmogorov–Smirnov test), we used a nonparametric statistics method for the correlation analyses, Spearman’s rank-order correlation coefficient. Then, we conducted a partial correlation analyses by controlling confounding factors to examine the specific relationships between WM and each motor function. WM data were averaged across face and location because we had originally focused on the relationship between “visually-encoded WM” and TUG.

#### fMRI Data

The fMRI data were analyzed using Statistical Parametric Mapping 8 (SPM8; Wellcome Department of Imaging Neuroscience, London, UK) implemented in MATLAB R2010b (The Mathworks, Natick, MA, USA). All of the functional images were spatially realigned to the first functional image to correct for head motion. Resulting volumes were normalized to a standard EPI template based on the Montreal Neurological Institute (MNI) reference brain (re-sampled voxel size 2 mm × 2 mm × 2 mm). The normalized images were smoothed using an isotropic 8 mm full width at half maximum Gaussian kernel. A high-pass filter of 1/128 Hz was used to remove low-frequency noise and an AR (1) model was used to correct temporal autocorrelation.

The fMRI data were analyzed using the blocked design. Activated voxels in each experimental condition were identified using a statistical model containing a boxcar function convolved with a canonical hemodynamic response function. The experimental conditions consisted of the following: (1) 1-back task; (2) 0-back task; and (3) rest. To maximize the statistical power, we analyzed the fMRI data collapsing across face and location WM tasks. Linear contrasts were used to obtain participant-specific estimates for each effect.

The correlation analyses of the fMRI activation (beta value) with given parameter of interest (TUG performance time) were performed at a second level through applying one sample *t*-tests to the first level *t*-maps resulting from the linear parametric modulation as implemented in SPM8. For 2 clusters, where significant correlations were found, we produced plots to show how TUG performance time was related to the activation during the WM task. The statistical threshold was set at *p* < 0.001 (uncorrected for multiple comparisons, cluster size > 10 voxels) for all of those analyses.

## Results

### Participant Characteristics

Table 1 summarizes the basic characteristics of the all participants analyzed. Because we set strict criteria to neutralize the general cognitive decline related effect, the mobility scores were a little better than expected for their age matched community-dwelling counterparts, but were within 95% CI range (Bohannon, 2006; Bohannon and Williams Andrews, 2011).

**Table 1:** **Characteristics of the participants (*N* = 32)**.

Age	73.06 (4.83)
Gender (*N* of women)	12
Education	12.88 (2.03)
MMSE	28.81 (1.00)
10MWT	7.8 (0.84)
TUG	6.36 (1.13)

### Behavioral Data

WM performances were analyzed by a 2 (cognitive load: 0- and 1-back) × 2 (stimuli: face and location) ANOVA. It found significant main effects of cognitive load (*F*_(1,31)_ = 21.693, *p* < 0.01), stimuli (*F*_(1,31)_ = 22.97, *p* < 0.01) and significant interaction between them (*F*_(1,31)_ = 16.93, *p* < 0.01). The results indicated that the 1-back task was more difficult than the 0-back task, and that the face stimuli were more difficult than the location stimuli. A *post-hoc* multiple comparison analysis showed that a simple main effect of stimuli was not significant at the level of the 0-back load (*F*_(1,62)_ = 0.01, n.s.) but was at the 1-back cognitive load (*F*_(1,62)_ = 39.17, *p* < 0.01). Because of its simplicity, WM performances were high enough to produce a ceiling effect for location WM (mean accuracy + 1SD > 1); however, the performance of face WM was lower than that of location WM and was consistent with previous studies (Leonards et al., 2002; Kawagoe and Sekiyama, 2014).

**Figure 1:**
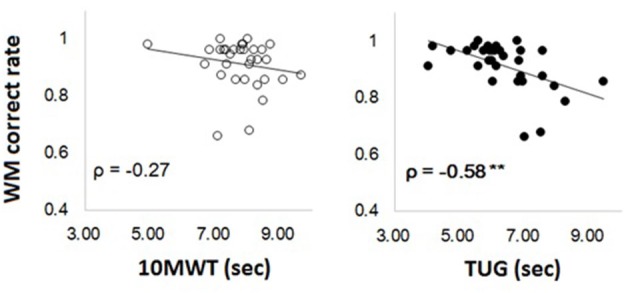
**Scatter plots for the correlations between Working Memory (WM) (average score of face and location WM) and time needed to perform 10MWT and timed up and go test (TUG) (left for 10MWT and right for TUG)**.

In our prior study, we tested the WM’s relationship only to the TUG performance. Figure 1 showed the scatter plots and correlation coefficients between WM performance and the two tasks of mobility, which indicates the difference between simple walking and TUG in terms of correlation with WM. After calculating with the spearman’s rank order correlation coefficient (rho: ρ), a significant correlation was found only between WM and TUG (*ρ* = −0.58, *p* < 0.01), but not between WM and 10MWT (*ρ* = −0.29, n.s.).

### fMRI Data

We first examined brain activation for WM by the contrast of 1-back vs. 0-back conditions for a combined data for location and faces in search for regions showing correlation with TUG performance in a whole brain analysis. However, we could not find any region under the present threshold (*p* < 0.001, uncorrected for multiple comparisons, cluster size > 10 voxels). Presumably because of the older adults’ characteristics, a lot of areas were significantly activated in the 0-back task. Consistent with a recent study showing prefrontal activation for a simple reaction task only in older adults (Berchicci et al., 2014), the present 0-back task may have imposed much attentiveness in the older adults as indicated by the prefrontal activation. If so, the contrast of 1-back vs. 0-back may have caused an underestimation of executive-related activation.

**Figure 2:**
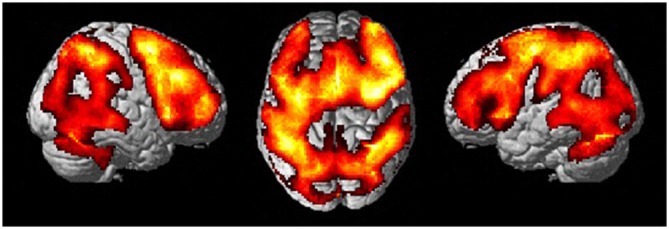
**Activation maps for 1-back vs. rest contrast.** Stimulus type (across for faces and locations) was collapsed to maximize the statistical power.

**Figure 3:**
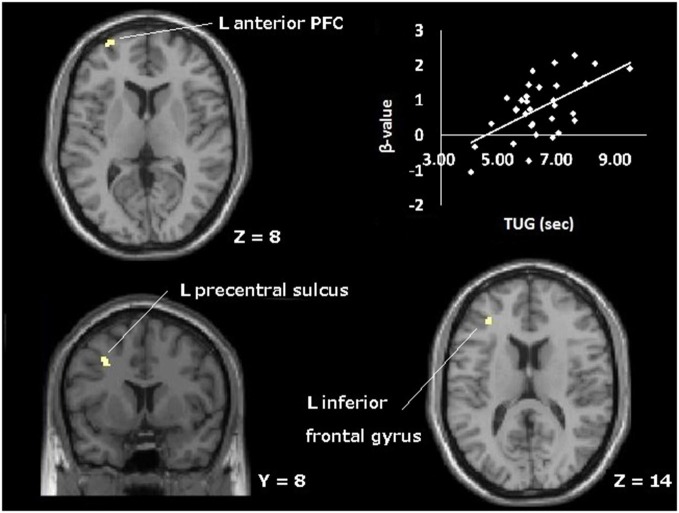
**Topographical map of areas showing significant positive correlation between brain activation during WM (1-back vs. rest contrast) and time needed to perform TUG.** The plot represents the correlation at the left anterior prefrontal cortex; *L*, left; *PFC*, prefrontal cortex.

Thus, we focused on the contrast between 1-back and rest conditions as a measure of activation for WM. Figure 2 shows the significant activation at the contrast of 1-back vs. rest. Although a large are of the regions was significantly activated, these regions include typical areas for WM. The areas where robust activation was reported in the WM task were included in the contrast without exception: lateral premotor cortex, dorsal cingulate, medial premotor cortex, dorsolateral and ventral prefrontal cortex (PFC), frontal poles, medial and lateral posterior parietal cortex (Owen et al., 2005). Then, we carried out the correlation analyses. Within the 1-back vs. rest contrast, some regions were found to be positively correlated with the time needed for TUG (Figure 3, Table 2). The regions were left precentral sulcus (BA6), left middle frontal gyrus (BA10) and left inferior frontal gyrus (BA45). In these left frontal regions, increased brain activation was observed for those who needed more time to perform the TUG.

**Table 2:** **Location in the MNI space of the peak voxel showing positively negatively correlated regions; L, left; R, right**.

Hemisphere structure	BA	Cluster	*T*-value	MNI coordinates		
				*x*	*y*	*z*
*Positively correlated with time needed for TUG*						
L precentral sulcus	6	63	7.27	−34	2	38
L middle frontal gyrus	10	36	5.73	−32	60	8
L inferior frontal gyrus	45	14	5.04	−30	40	14
*Negatively correlated with time needed for TUG*						
R precentral gyrus	6	10	8.94	54	10	42
R cerebellum	–	61	7.66	4	−58	−36
R thalamus	–	104	7.23	18	−26	12
R putamen	–	17	6.38	28	−28	4

**Figure 4:**
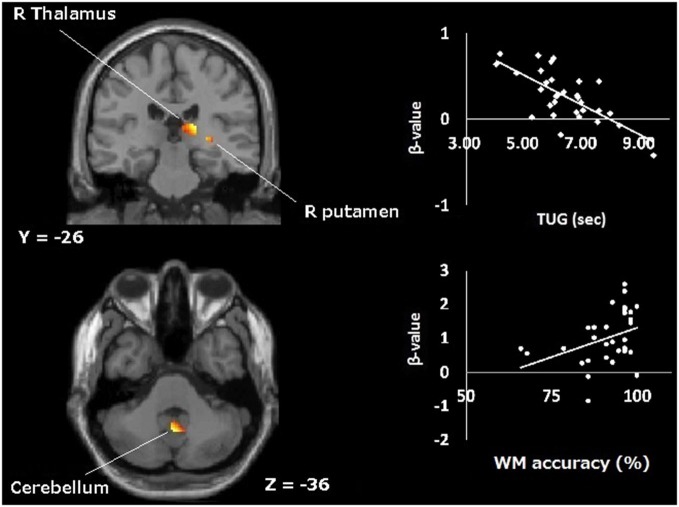
**Topographical map of areas showing significant negative correlation with time taken for TUG during WM (1-back vs. rest contrast).** The upper plot represents the correlation at the right thalamus. The lower plot represents the correlation between activation during visual WM and its performance at cerebellum; *R*, right.

Additionally, that contrast revealed regions with significant negative correlations with the time needed for the TUG (Figure 4, Table 2), indicating that less activation was observed in these regions for those who needed more time to perform the TUG. The areas were right thalamus, right cerebellum, right putamen and right precentral gyrus. The regions in which activation decreased with declined TUG performance were mostly subcortical structures including the basal ganglia and cerebellum in the right hemisphere.

Finally, we calculated the correlation between activation level (beta-value) in these TUG-correlated areas and WM performances (1-back task). Spearman’s rho between averaged beta-value and WM performance was −0.28 (*p* = 0.12) for the regions with positive correlations, and 0.46 (*p* < 0.01) for the regions with negative correlations.

## Discussion

The present study aimed to elucidate the neural correlates of WM-mobility association by using fMRI. The experimental design was determined based on the prior finding that visually encoded WM is associated with mobility. We assumed one of the compensation hypotheses (CRUNCH) in this study, which may predict “lower-mobility older adults would show a higher level of brain activation when the task is very easy”. We investigated the brain regions for which the activation level for WM significantly correlated with the TUG performance. Indeed, positive and negative correlations were found. We discuss our results and their implications as well as some limitations below.

Consistent with previous findings (Leonards et al., 2002; Kawagoe and Sekiyama, 2014), the task difficulty of WM for face compared to that for location was observed in older adults even in the 1-back task which is thought to be the easiest task in WM. Correlation analysis found a significant relationship between WM and TUG while we found no significant correlations between WM and the simple walking test. The correlation coefficient between TUG and WM was twice as large as the coefficient between 10MWT and WM. It indicated that the relationship with WM was stronger in the functional mobility task, which needs more complex motor control, than the simple walking task. Previous studies support this result (Ble et al., 2005; Donoghue et al., 2012) although other studies indicate that simple walking is a good measurement of older adults’ function for living (Yogev-Seligmann et al., 2008; Bohannon and Williams Andrews, 2011; Peel et al., 2013). The difference might be because the 10MWT needs visual updating less than the TUG, and which is a critical role for visual WM.

Significant correlations between the brain activation during WM and TUG performance were confirmed in this study. A previous study suggests that highly fit older adults could cope with the imaginary walking task by less PFC activation (Godde and Voelcker-Rehage, 2010). So, we could guess that if brain activation during TUG had been measured, it would have been correlated with TUG performance especially in PFC. As expected, some frontal area’s activations were positively correlated with the time needed for TUG (positively correlated regions; PCR). These PCR have been implicated for WM by meta-analyses (Owen et al., 2005; Rottschy et al., 2012), and in visuospatial WM studies for anterior PFC (Ranganath et al., 2003; Leung et al., 2005). As already mentioned in the introduction, we can interpret the activation data based on CRUNCH. The PCR was expected by the present assumption because the worse TUG performers activated the frontal areas more. The left lateralization of PCR might be owing to the nature of the task. The compensatory activations in the PFC have been observed in the left hemisphere when the performed task was achieved originally (e.g., in young adults) by right hemispheric activation (Cabeza et al., 2002; Cabeza, 2002).

Meanwhile, activations of some regions were negatively correlated with time to perform TUG, significantly (negatively correlated regions; NCR). The NCR could not be simply explained by the assumption of CRUNCH. NCR activities were weakened with TUG decline. It is conceivable that those NCR regions are not able to resist age-related deterioration. NCR were mainly subcortical structures including parts of the basal ganglia (putamen), thalamus and cerebellum. The thalamus is involved in both cognitive control (Van der Werf et al., 2003) and motor control as a node of the pathways from the cerebellum to the motor areas, from the basal ganglia to the motor areas, and from the superior colliculus to the frontal eye field (Sommer, 2003). This region, together with the putamen, is part of some “circuits” relating to cognitive and motor function (Alexander and Crutcher, 1990). Also the cerebellum is a representative area for motor control and it is also involved in WM processing as a central executive system (Hautzel et al., 2009). The hypo-activation might be signs of the older brains’ deterioration. As evidence of this, the correct rates of WM were positively correlated with those NCR activation (Figure 4, lower plot). Collectively, the results showed that the worse TUG performers had less activated NCR and more activated PCR during WM. Additionally, the right-lateralized activation of NCR would be explained by the types of material used in the WM task. We used nonverbal stimuli, human face and random location of a dot, which was difficult to verbalize. Nonverbal stimuli yielded right-lateralized activations in medial temporal regions during episodic memory (Kelley et al., 1998), and in occipital regions during WM (Smith et al., 1996).

The compensation hypothesis postulates that older participants have to rely on more prefrontal control than young adults to compensate for the deterioration in low level processing in motor control (Heuninckx et al., 2005; Noble et al., 2011; Berchicci et al., 2014; for review, Seidler et al., 2010) and in a variety of cognitive tasks (Grady et al., 1994; Davis et al., 2008). The “deterioration”, however, remains hypothetical, being assumed as the flip side of compensation, such as neuronal and/or structural age-related change (Greenwood and Parasuraman, 2010; Seidler et al., 2010). Few studies have actually demonstrated the degraded activation of the subcortical regions. The present results of NCR clearly demonstrate such declining as degraded activations, and those were considered to be the overlapping regions, needed in WM and mobility control. We consider that the present results of NCR and PCR exhibited a common dysfunction—compensation pattern across the WM and TUG. Participants who suffer the deterioration of subcortical areas (NCR) would activate more prefrontal “attentional” regions (PCR) for executing, at least, WM and mobility tasks. For the compensational role of PCR, in agreement with the current study, the inferior frontal gyrus (BA45) was reported as a region which activated for compensation in episodic retrieval and visual perceptual tasks (Davis et al., 2008) and the middle frontal gyrus (BA10) was also observed as such a compensation-related region in a visual matching task (Grady et al., 1994), although those studies reported other compensatory activations as well. About the compensation, we had expected that parietal regions would be related to the compensational activation but we could not find such activation. Parietal areas were reported as the regions reflecting the number of visual representation held in our mind (Todd and Marois, 2004). In our experiment, the small amount of information to be held (one item) may have not been enough to activate the parietal area to significantly correlate with motor performance. In sum, these results suggest that heightened PCR activations were to protect older adults’ behavioral ability from age-related deterioration in the NCR regions. Perhaps, the reason why physical exercise benefits older adults physically and cognitively may be that the exercise can activate the degraded regions such as the NCR as well as reinforce efficiency of the compensational regions such as the PCR (e.g., Colcombe et al., 2004).

The present results provide insights into the relationship between WM and functional mobility in older adults, which indicates the existence of common dysfunction—compensation pattern of brain activation. Nevertheless, we should mention some limitations in this study. First, TUG produced small variance and high average scores compared to normative data of healthy older adults in prior studies (Shumway-Cook et al., 2000; Kawagoe and Sekiyama, 2014). This is because we set comparatively high MMSE criteria for exclusion to control the effect of general cognitive function, so that only the high-functioning older adults were included in the analysis. Then, the result of the TUG score was similar to a previous study of well-functioning older adults (Berryman et al., 2013) and the simple walking speed fitted in the meta-analyzed data of healthy older adults (Bohannon and Williams Andrews, 2011). Thus, the generalization of the present finding to low-functioning older adults remains for future research. Second, the contrast data we used were 1-back vs. rest condition, although we originally intended to use the 0-back condition as a baseline. In view of prior studies suggesting that older adults need additional activities including PFC to perform elementary tasks (Berchicci et al., 2014), the 0-back task might be not the most suitable baseline condition for older adults although it is commonly used for younger adults. The contrast of 1-back vs. rest had a risk of including the response-related activation. However, we consider that such a possibility is low because motor related primary areas such as M1 were not included in the results of the WM-TUG correlation analyses. The present paradigm could have elucidated the common dysfunction—compensation pattern of activation between cognition and motor control in older adults.

In conclusion, we suggest the age-related change of the functional brain activation for both cognitive and motor tasks, by utilizing the relationship between visually encoded WM and TUG in healthy older adults. The common change in functional brain activation might be built upon the general prefrontal compensation response to the age related deterioration in the subcortical regions. Although there are some limitations noted above, this study may be the first report to uncover older adults’ common neural characteristics to cognitive and motor domains as the dysfunction—compensation pattern of brain activation.

## Author Contributions

TK, MY, SN, MY, KS, SY, NA, and YO constructed the conception and design of the study. Data collection and analyses were mainly done by TK, MS, SN, NA, YO and RN. Drafting of the article was done by TK and KS but revised by the other authors, MS, SN, MY, SY, NA, YO, and RN.

## Conflict of Interest Statement

The authors declare that the research was conducted in the absence of any commercial or financial relationships that could be construed as a potential conflict of interest.
